# The First Survey of Forensically Important Entomofauna Collected from Medicolegal Autopsies in South Korea

**DOI:** 10.1155/2015/606728

**Published:** 2015-06-22

**Authors:** Sang Eon Shin, Hyun Ju Lee, Ji Hye Park, Kwang Soo Ko, Yu-Hoon Kim, Kyung Ryoul Kim, Seong Hwan Park

**Affiliations:** ^1^Department of Legal Medicine, Korea University College of Medicine, 73 Inchon-ro, Seongbuk-gu, Seoul 136-705, Republic of Korea; ^2^National Forensic Service, 10 Ipchun-ro, Wonju-si, Gangwon-do 220-170, Republic of Korea

## Abstract

Forensic entomology applies insect evidence to legal problems such as the estimation of minimum postmortem interval (mPMI). For this purpose, knowledge of the insect fauna that are attracted to human cadavers in each geographic region is a prerequisite. Despite many studies investigating the insect fauna attracted to meat, there has been no survey of the entomofauna on human cadavers in the East Asian temperate climate zone, particularly in Korea. Therefore, this study reports the entomofauna collected from medicolegal autopsies in northeastern Seoul and its suburbs. Insect samples were collected from 35 medicolegal autopsies in 2010, 2011, and 2013. Molecular and morphological methods were utilized for taxonomic identification. Among 1398 individual samples belonging to 3 orders, 13 families, 18 genera, and 32 species, the dominant family and species were Calliphoridae and *Lucilia sericata*, respectively. Despite its limited scale, this study provides a snapshot of the general entomofauna that are attracted to human cadavers in this region.

## 1. Introduction

Medicolegal entomology, which applies insect evidence to crime scene investigations, is one of the main fields of forensic entomology [1]. Traditional methods that use early postmortem phenomena, such as postmortem body cooling, livor mortis, and rigor mortis, to estimate the postmortem interval (PMI) become invalid once postmortem phenomena such as autolysis and putrefaction occur [2]. Evidence derived from insects that visit a dead body is an alternative strategy that can be used to estimate the minimum PMI (mPMI) once the early postmortem phenomena become invalid. To estimate mPMI using insect evidence, knowledge of the entomofauna that are attracted to dead bodies in a specific geographic region is critical, as is the knowledge of the growth rates of larvae of dominant carrion insects [1]. Therefore, several researchers have investigated the entomofauna that are attracted to human cadavers in various geographic regions [3–5]. In Korea and neighboring East Asian countries within a similar temperate climate zone, the DNA of dipteran species that are attracted to pork liver baits or pig carcasses has been analyzed for species identification [6–11]. However, there has been no survey of carrion insects visiting human cadavers in the temperate climate zone of East Asia despite medicolegal autopsies being quite actively performed. In this study, 35 medicolegal autopsies of insect infestation were investigated in the northeastern area of Seoul and its suburbs over three years to determine the forensically important entomofauna of this region.

## 2. Materials and Methods

### 2.1. Sampling

Insect infestation was observed in 35 medicolegal autopsies in northeastern Seoul and its suburbs during 2010, 2011, and 2013. With the consent of the forensic pathologist who performed the autopsy, about 40–50 individual insects were collected from each cadaver. When there are numerous maggots and small numbers of other insects, maggots and other insects were collected randomly and totally, respectively. If there were very small numbers of individual insects on a cadaver, all of the individual insects were collected. Approximately, half of the live maggots collected were preserved in an 80% ethanol solution at 4°C and the other half of the live maggots collected were sent to the incubation chambers for rearing and morphological taxonomic identification. Nondipteran samples were immediately preserved in 80% ethanol solution without rearing. All the samples immersed in 80% ethanol solution were immediately sent to a freezer (−20°C). Although the best method of killing and preservation of live maggots is treatment in boiling water and then transfer to 80% ethanol solution to avoid browning and shrinkage of maggots [12, 13], the process of immersion in boiling water was omitted because the purpose of the preservation was merely DNA barcoding.

### 2.2. Taxonomic Identification

Morphological features of adult individuals were observed under an SZX10 stereomicroscope (Olympus Corporation, Tokyo, Japan). Maggots used for rearing were taxonomically identified to the species level after emergence [14–17]. After initial identification at a family level on the basis of the shape of the posterior spiracles, a maggot sample was used for DNA barcoding. All the adult beetles and beetle larvae were taxonomically identified on the basis of their morphological features to the species and the family level, respectively [18–21].

### 2.3. DNA Barcoding of Maggots

DNA barcoding of maggots was performed utilizing the mitochondrial cytochrome c oxidase subunit I gene (*COI*). Polymerase chain reaction was performed mainly to amplify the barcoding regions located within the upstream third portion of* COI*. Primer sequences, which were adopted from a previous study, are listed in Table 1 [6]. After automatic sequencing using an ABI Prism 3720xl genetic analyzer (Applied Biosystems, Foster City, CA, USA), nucleotide sequences were aligned and compared with preexisting nucleotide sequences using MEGA6 Software [22].

### 2.4. Rearing Conditions for Maggots

Because the identification of maggot species using morphologic features is difficult, specimens were reared to the adult stage inside incubation chambers under constant conditions (25°C, relative humidity 70%). The light and dark ratio of each chamber was set at 16 : 8. Live maggots were placed on 5-6 g of fresh pork liver in a plastic cylinder measuring 95 mm in height and diameter with an adequate amount of sawdust under the pork liver.

## 3. Results and Discussion

**Figure 1:**
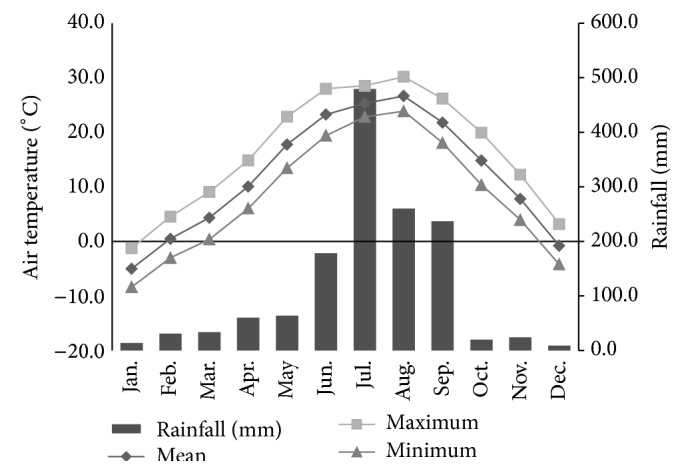
A climograph of mean, maximum, and minimum air temperatures (°C) and rainfall (mm) at Seoul weather station (37°34′N; 126°58′E) in 2010, 2011, and 2013 demonstrates hot, humid summers and cold, dry winters.

**Figure 2:**
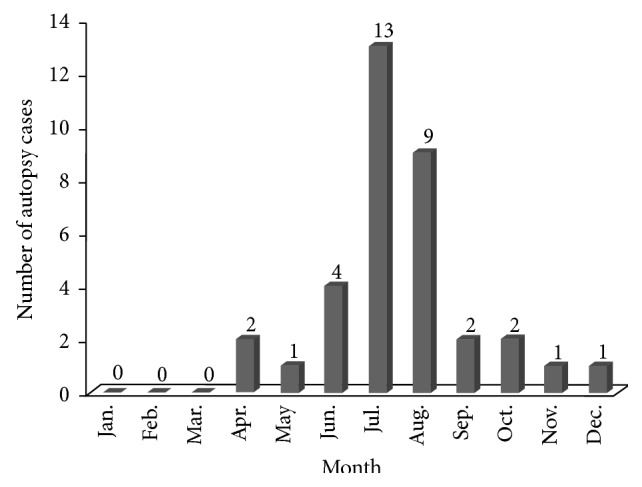
Medicolegal autopsy cases showing insect infestation in the study region in 2010, 2011, and 2013 were most frequent in the summer (June, July, and August).

**Figure 3:**
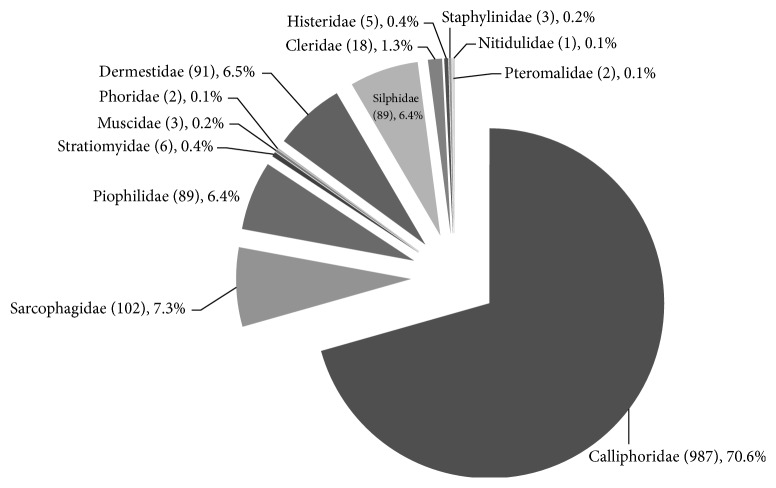
Individual numbers of collected insects in each family among 1398 insect samples.

Weather records for the study period confirmed that northeastern Seoul belongs to the temperate climate zone having humid, hot summers and dry, cold winters with intervening moderate springs and falls (Figure 1). Insect infestation of human cadavers was most common (26 autopsies) in summer (June, July, and August) and was not observed from January to March (Figure 2). Among 1,398 individual samples, a total of 3 orders, 13 families, 18 genera, and 32 species were identified. The three orders identified were Diptera (1,189 individuals, 85.1%), Coleoptera (207 individuals, 14.8%), and Hymenoptera (2 individuals, 0.1%). The most common families, Calliphoridae (987 individuals, 70.6%) and Sarcophagidae (102 individuals, 7.3%), occupied 77.9% (Figure 3).* Lucilia sericata* was collected from 22 of 35 autopsies (62.9%) and showed the highest relative frequency, followed by* Sarcophaga peregrina* (7 autopsies, 11.4%). Among beetles,* Dermestes maculatus* was the most common species (4 autopsies, 11.4%). The entomofauna observed in the present study is summarized in Table 2.

It is well known that the insect fauna changes with geographic region and climate. Studies of the insect fauna associated with human cadavers are fundamental to forensic entomology. In experiments with pig carcasses, it is difficult to collect insects from various regions unless the experiments are performed in many different locations. However, because the locations of unusual deaths are varied, samples from a variety of regions are included in the present study. As the research of Cainé et al. in Portugal [5], the present study utilized molecular barcoding as well as morphological identification [14–21] to ensure confident species identification. The present study showed a strong preference of incidence to summer, whereas a previous study in Thailand, belonging to tropical rainforest, did not display any seasonal preference [23].

The dominant species in the present study,* Lucilia sericata*, showed a strong preference for indoor environments, which has been demonstrated in previous studies [24].* Calliphora vicina* and* Calliphora lata* showed bimodal seasonal occurrence in spring and autumn. According to a previous work by Park in a more southern part of Korea, these cryophilic species tend to prefer cool seasons or high altitude in summer [25].* C. vicina*, a common species in the Holarctic region, is particularly common in Northern Europe [26]. Unlike* C. vicina*, the geographic distribution of* C. lata* is known to be limited in the far-east Asia [27]. Sarcophagidae species only occurred during summer. Interestingly, Muscidae species occurred only once in the present study:* Hydrotaea obscrifrons*, here recorded for the first time in Korea. This* H. obscrifrons* maggot was collected from a moderately mummified body abandoned inside a car at an underground parking lot in the downtown. A previous study showed that flies belonging to the genus* Ophyra* (=* Hydrotaea*) mainly appear at later stages during ammoniacal fermentation, as shown in the present study [26].* Piophila casei* (family Piophilidae), which is a potential indicator of the advanced stage of decay [17, 28], was found from two cadavers showing adipocere. Although this synanthropic species was expected to exist in Korea, this was the first official identification of this species in Korea. Another Piophilidae species,* Parapiophila vulgaris*, was found to coexist with* P. casei* in one case. Maggots of* Hermetia illucens* (family Stratiomyidae), which are known to feed on a wide variety of decaying organic materials [29], were found in a severely putrefied body abandoned in a room.* Megaselia scalaris* (family Phoridae) pupae were found in a closed room. Because species belonging to the family Phoridae are small in size and can easily enter closed spaces [30], these flies are potential indicators for mPMI estimation in cases where other flies have poor access to the body due to its presence in a closed environment [30]. Coleoptera species appeared entirely in the summer except for an individual* Dermestes haemorrhoidalis* that was found in a greenhouse in December (Table 2). In Sukontason et al.'s study in Thailand,* D. maculatus*, the dominant beetle species in the present study, was the only beetle species collected [23].* Dermestes* spp. are the first visitors of Mégnin's third wave [31]. Silphidae species were observed 4 times exclusively in the forest cases.* Necrodes littoralis* (subfamily Silphinae) and* Nicrophorus quadraticollis* (subfamily Nicrophorinae) were identified. Although some species in the family Silphidae are known to be remarkable indicators during fresh and bloating stages of decomposition [1], in the present study,* N. quadraticollis* was collected from the skull of a skeletonized body. Only eight individuals were collected for families Histeridae, Staphylinidae, and Cleridae known to feed on maggots [31]. Because the sample size of the present study was limited, more extensive sampling is required to characterize the forensically important beetle fauna in Korea.

In conclusion, although all insect species expected to be observed during a medicolegal autopsy could not be fully covered due to the limited scale, the present study provides a general overview of forensically important insect species in Korea. Further studies over wider geographical regions in Korea are required to establish more faithful data regarding forensically important entomofauna.

## Figures and Tables

**Table 1:** Primer sequences used for the amplification and sequencing of dipteran *COI* genes.

Name	Sequence	Binding site
F1	5′-CCTTTAGAATTGCAGTCTAATGTCA-3′	tRNA-cysteine
F2	5′-GGAGGATTTGGAAATTGATTAGTTCC-3′	220–245 on *COI *
F3	5′-CTGCTACTTTATGAGCTTTAGG-3′	1000–1022 on *COI *
R1	5′-CCTAAATTTGCTCATGTTGACA-3′	2–23 on *COII* ^*∗*^
R2	5′-CAAGTTGTGTAAGCATC-3′	1327–1343 on *COI *
R3	5′-CCAAAGAATCAAAATAAATGTTG-3′	688–710 on *COI *

^*∗*^ *COII*: cytochrome c oxidase subunit II gene.

**Table 2:** Numbers of medicolegal autopsy cases where each insect species occurred.

Scientific names	Location			Months									Total	Frs (%)
	I	O	F	Apr.	May	Jun.	Jul.	Aug.	Sep.	Oct.	Nov.	Dec.		
Order Diptera														
Family Calliphoridae														
*Lucilia sericata *(Meigen, 1826)	21	1		2	1	2	8	4	2	1	1	1	22	62.9
*L. caesar *(Linnaeus, 1758)			1					1					1	2.9
*L. illustris *(Meigen, 1826)			1					1					1	2.9
*Calliphora lata *(Coquillett, 1898)	2				1						1		2	5.7
*C. vicina *Robineau-Desvoidy, 1830	2			1	1								2	5.7
*Phormia regina *(Meigen, 1826)	4	2	1			1	3	2					6	17.1
*Chrysomya megacephala *(Fabricius, 1794)	2							2	2				2	5.7
*C. pinguis *(Walker, 1858)	2		2					2	1	1			4	11.4
Family Sarcophagidae														
*Sarcophaga dux *Thomson, 1869	1							1					1	2.9
*S. crassipalpis *Macquart, 1839	2					1	1						2	5.7
*S. peregrina* Robineau-Desvoidy, 1830	4		3			2	2	3					7	20.0
Family Muscidae														
*Hydrotaea obscrifrons *(Sabrosky, 1949)		1					1						1	2.9
Family Piophilidae														
*Piophila casei *(Linnaeus, 1758)	1	1					1	1					2	5.7
*Parapiophila vulgaris *(Fallén, 1820)	1						1						1	2.9
Family Stratiomyidae														
*Hermetia illucens *(Linnaeus, 1758)	1						1						1	2.9
Family Phoridae														
*Megaselia scalaris *(Loew, 1866)	1							1					1	2.9
Order Coleoptera														
Family Dermestidae														
*Dermestes ater *(DeGeer, 1774)			1			1							1	2.9
*D. frischi *Kugelann, 1792		1	1			1		1					2	5.7
*D. haemorrhoidalis *Küster, 1852	2	1						2				1	3	8.6
*D. maculatus *(DeGeer, 1774)	3	1					2	2					4	11.4
*D. tessellatocollis *Motschulsky, 1860	1						1						1	2.9
Family Silphidae														
*Necrodes littoralis *(Linnaeus, 1758)			1			1							1	2.9
*Necrodes* sp.			2				1	1					2	5.7
*Nicrophorus quadraticollis *Portevin, 1903			1				1						1	2.9
Family Histeridae														
*Saprinus *sp. 1			1			1							1	2.9
*Saprinus* sp. 2	1						1						1	2.9
Family Staphylinidae														
*Philonthus cyanipennis *(Fabricius, 1793)			1			1							1	2.9
*P. longicornis *Stephens, 1832	1						1						1	2.9
*Philonthus *sp.			1				1						1	2.9
Family Nitidulidae														
*Omosita *sp.	1						1						1	2.9
Family Cleridae														
*Necrobia rufipes *(DeGeer, 1775)	2	1					1	2					3	8.6
Order Hymenoptera														
Family Pteromalidae														
*Nasonia vitripennis *(Walker, 1836)	1						1						1	2.9

Number of collected species	21	8	13	2	3	9	18	15	3	2	2	2	32	

I, O, and F stand for indoor, outdoor, and forest, respectively. “Frs” means frequencies.
